# Serum VEGF-A concentrations in patients with central nervous system (CNS) tumors

**DOI:** 10.1371/journal.pone.0192395

**Published:** 2018-03-28

**Authors:** Agnieszka Nowacka, Wojciech Smuczyński, Danuta Rość, Kamila Woźniak—Dąbrowska, Maciej Śniegocki

**Affiliations:** 1 Department of Neurosurgery, Neurotraumatology and Paediatric Neurosurgery, Nicolaus Copernicus University Collegium Medicum in Bydgoszcz, Poland; 2 Department of Neurotraumatology, Nicolaus Copernicus University Collegium Medicum in Bydgoszcz, Poland; 3 Department of Pathophysiology, Nicolaus Copernicus University Collegium Medicum in Bydgoszcz, Poland; University of Navarra, SPAIN

## Abstract

Angiogenesis plays an essential role in tumors development. In case of central nervous system tumors, the most important role in this process plays VEGF-A. The purpose of this study was to determine the plasma concentration of this agent in patients treated surgically because of intracranial tumors. The study involved 48 adult patients, both sexes, treated surgically because of a brain tumor. The control group consisted of 50 adult volunteers of both sexes, without cancer diagnosis. Based on the studies, it was found that serum VEGF-A levels before surgery are higher in patients with central nervous system tumors (10.39–150.57 pg/ml, median 41.70 pg/ml) than in non-cancer patients (0.00–130.77 pg/ml, median 22.56 pg/ml). The association between serum VEGF-A level and malignancy and histological type of intracranial tumor has not beed confirmed. The highest average preoperative serum VEGF-A level was found in patients with low grade gliomas, slightly lower (close to each other) in those with high grade gliomas and meningiomas, while the lowest level was characteristic for metastatic tumors. High variation in results was observed in patients with low grade gliomas (52.56 pg/ml)—higher than those reported in patients with high grade gliomas (32.38 pg/ml). In the rest types of tumors the differentiation was similar and oscillated within 23.08–27.50 pg/ml.

## Introduction

Angiogenesis is a multi-factorial and multi-stage process leading to the formation of new blood vessels from existing ones. It plays a crucial role in the development of tumor—it determines its growth and metastasis [1]. There are many proangiogenic substances. The most important and at the same time the best known factor affecting both physiological and pathological angiogenesis is vascular endothelial growth factor (VEGF) [2], which produces its biological effect by binding with high-affinity receptors in vascular endothelial cells—VEGFR-1 (vascular endothelial growth factor receptor 1, Flt-1) and VEGFR-2 (vascular endothelial growth factor receptor 2, KDR / Flk-1), belonging to the family of tyrosine kinase receptors [3]. At present, the most important role in neoangiogenesis of central nervous system tumors seems to play VEGF-A with its VEGFR-2 receptor. This factor is genetically stable over time and its expression, as the only factor of angiogenesis, in the tumor development process is continuous.

The important role of vasculitropin in the process of solid tumors’ neoangiogenesis is associated with its increased concentration in body fluids—high expression of VEGF is associated with increased tumor aggressiveness (rapid growth, metastasis) and the same—poor prognosis. Elevated levels of VEGF and VEGF mRNA, both in tumor tissue and plasma, serum or urine, have been reported in many cancers, including colorectal, stomach, breast, non-small cell lung cancer, prostate, kidney, bladder [4].

Due to increasing interest of scientists of neoangiogenesis in central nervous system tumors, more and more methods are available to monitor this process. The highest correlation level to histopathological assessment of central nervous system tumors' angiogenesis, among cytokines, has serum VEGF-A level determined by ELISA (Enzyme-Linked Immunosorbent Assay). This is a test that does not require additional patient load, as it uses blood drawn routinely before and after planned surgery. At the same time, its execution can provide valuable information as to the degree of neoangiogenesis, and thus have a great prognostic significance. On this basis, it was considered intentional to determine serum VEGF-A concentration.

## Materials and methods

In order to conduct the research, the approval of the Bioethics Committee of Nicolaus Copernicus University Collegium Medicum in Bydgoszcz was obtained, its number being: KB-665/2009. The study involved 48 adult patients (M_b_ = 60.16) of both sexes (21 women and 27 men), treated surgically for solid brain tumor in the Department of Neurosurgery, Neurotraumatology and Paediatric Neurosurgery Nicolaus Copernicus University Collegium Medicum in Bydgoszcz, from February 2010 to April 2011. The diagnosis that lead to qualifying the patients for the examination was given based on interview, physical as well as neuroimaging examinations, and in the later stage it was confirmed with the post-surgical histopathological examination of the removed tumor. The patients who had disorders of consciousness that prevented them from giving their informed consent for taking part in the study were excluded from it.

The control group consisted of 50 adults (M_p_ = 59.16) volunteers of both sexes (24 women and 26 men), without tumor diagnosis, who did not underwent surgery in less than 30 days, negating occurrence of diabetes, hypertension and coronary heart disease, and do not taking any medicines on a regular basis.

All the people who were qualified for the study, after being informed of its subject, gave their written consent to taking part in it.

The test groups were determined as follows: b—study group, p—comparative group. Study groups did not show statistically significant differences in sex (p = 0.71) and age (M_b_ = 60.16; M_p_ = 59.16; t = 0.4265; p = 0.67). The youngest patient in the study group was 26 years old and the oldest 79 years, the standard deviation was over a decade (SD = 10.79). In the comparison group the youngest person was 28 years old and the oldest was 79 years old. The age difference in this group was slightly higher (SD = 12.53).

In the group of patients with cancer, the majority had grade III (27.083%) or IV (33.333%) tumors, according to the 4-grade WHO scale. Grade I and II was characteristic for 20.833% and 18.75% of patients, respectively.

Classification of malignancy was also made in a general way for: malignant neoplasms—III and IV degree according to WHO, which was diagnosed in 60.42% of patients, and non-malignant—I and II degree according to WHO in 39.58%.

The frequency of the occurrence of the particular tumor types was assessed by the division into the 5 general groups of intracranial tumors: high grade gliomas (HGG), low-grade gliomas (LGG), meningiomas, metastatic tumors and others (Antoni B type schwannoma, Antoni A type schwannoma and adenoma hypophysis). The majority of people of the studied group had glioma-type tumors–in total, they constituted 62.5% of the studied group. The type that prevailed the most often were HGG; they were found in almost half of the studied group (47.92%). 14.58% of the studied group had low-grade gliomas. The next type–meningiomas, was characteristic in every 6^th^ studied patient (18.75%). Metastatic tumors were characteristic for 12.5% of patients. The tumors from the last group were found the least often–in slightly more than 6% of the studied group.

## Methods

The study in the researched group was conducted according to the following scheme:

1. Gaining written, informed patient’s consent to take part in the study.
2. Collecting the first venous blood sample (2 ml), up to 24 hours before surgery.
3. Performing the planned surgery consisting in removing the brain tumor.
4. Collecting the second venous blood sample (2 ml), during the first 24 hours after surgery.
5. Histological assessment of the material gained from the surgically removed brain tumor.
6. Determination of serum VEGF-A concentration in blood samples.

### Determination of serum VEGF-A concentration

Venous blood was collected to a plastic tube with 3.2% sodium citrate, using a Vacuette® Vacuum Blood System from Greiner Bio-One. This procedure was done in accordance with the blood collection procedure valid in the Clinic.

Further laboratory studies, to assess serum VEGF-A in people in the study and control group, were performed in the Chair and Department of Pathophysiology Nicolaus Copernicus University Collegium Medicum in Bydgoszcz. The blood sample, delivered to the Chair immediately after being collected, was centrifuged in a cooling centrifuge at + 4°C for 20 minutes at 3000 rpm. Obtained citrate serum was partitioned into 200 μl aliquots to Eppendorf tubes. So processed material was frozen at -80°C. After all samples were collected, serum VEGF-A levels were determined by immunoenzymatic method ELISA, using the Human VEGF Immunoassay kit from R & D Systems. Reference values for serum concentrations of this agent have not been provided by the manufacturer.

### Statistical analysis

Statistical analysis of the collected material was carried out using the Statistica 9.0 package. Descriptive statistics and descriptive characteristics were used to describe the variables. For most of the tested variables, a deviation from the normal distribution was found, tested by Lillefors test (p <0.01), that’s why nonparametric statistics were used. Spearman's correlation coefficient matrix was used to examine relations among variables. The Man-Whitney U test was used examine the differences between the variables in the comparison of divalent variables, and to compare multivariant variables—Anova Kruskal-Wallis test. Comparison of the same variable between the two measurements was made using the Wilcoxon pair test. All results with the condition p <0.05, were considered statistically significant.

## Results

In the study group, the pre-surgery serum VEGF-A concentration ranged from 10.39 to 150.57 pg/ml (median 41.70 pg/ml), while in the comparative group 0.00–130.77 pg/ml (median 22.56 pg/ml)—Table 1.

**Table 1:** Descriptive statistics of tested variables—Serum VEGF-A in the study and comparative group.

	N	Mean	Median value	Minimum	Maximum	Standard deviation	Coefficient of variation
**Serum VEGF-A before surgery [pg/ml]–group b**	48	53.30	41.70	10.39	150.57	33.49	62.85
**Serum VEGF-A after surgery [pg/ml]—group b**	48	61.46	48.64	9.03	215.31	40.24	65.46
**Serum VEGF-A [pg/ml]–group p**	50	32.13	22.56	0.00	130.77	34.57	107.59

group b, study group; group p, comparative group.

A significant correlation was noted for serum VEGF-A before and after surgery (Table 2). The strength of this relationship was high, and the relationship was consistent—the level in the first measurement is related to the level in the second measurement.

**Table 2:** Spearman rank correlation for serum VEGF-A [pg/ml] before surgery and after surgery.

	serum VEGF-A [pg/ml] before surgery
**serum VEGF-A [pg/ml] after surgery**	0.574865*

* p < 0.05

VEGF-A, vascular endothelial growth factor—A.

The highest mean serum VEGF-A concentration (Table 3) was found in patients with LGG, slightly lower (close to each other) concentration in those with HGG and meningioma, and the lowest one was characteristic for metastatic tumors. Characteristic was also a high variability in LGG patients (52.56 pg/ml), which was higher than that the one reported in patients with HGG (32.38 pg/ml). In the rest types of tumors the differentiation was similar and oscillated within 23.09–27.50 pg/ml.

**Table 3:** Descriptive statistics—Serum VEGF-A [pg/ml] concentrations before surgery, depending on the tumor type.

Type of tumor (by 5 general groups)	N	Mean	Standard deviation	Minimum	Maximum
**Metastatic tumors**	6	40.78	24.39	10.39	74.87
**Meningiomas**	9	51.20	27.50	16.17	88.85
**Other**	3	46.00	23.09	19.35	59.86
**LGG**	7	69.58	52.56	20.41	150.57
**HGG**	23	53.38	32.38	10.86	120.17
**Total**	48	53.30	33.49	10.39	150.57

HGG, high grade gliomas; LGG, low grade gliomas.

In the first step, obtained results were analyzed to verify whether patients with central nervous system tumors has serum VEGF-A concentrations higher than those without tumor. Mann-Whitney U test verified the significance of the differences between the study and the comparison group (Table 4). On the basis of this, the difference between the results in the group with and without tumor was confirmed. Although ranks indicate higher rates in people with intracranial tumors, it is not sufficient to determine the level of difference.

**Table 4:** Mann-Whitney U test of differences between the study and the comparison group.

	Rank—b	Rank—p	U	Z	p
**Serum VEGF-A before surgery [pg/ml]**	2872.50	1978.50	703.50	3.52492	0.00042

b, study group; p, comparison group; VEGF-A, vascular endothelial growth factor—A.

People with brain tumor have a higher VEGF-A concentration—the median exceeds 41.70 pg/ml, while in the comparison group it is 22.56 pg/ml. Despite similar variability, the range of results in the comparison group ranges from 0 to 130.77 pg/ml, while in patients with tumors—10.39 to 150.57 pg/ml.

In the second step, obtained results were analyzed for correlation between VEGF-A and histological type and grade of malignancy according to WHO. For this purpose the relationship was determined between the VEGF-A and the histological type of the tumor according to WHO and malignancy grade on a malignant (WHO III and WHO IV) / non-malignant (WHO I and WHO II) scale—Table 5.

**Table 5:** Spearman rank correlation for serum VEGF-A concentration [pg/ml] before surgery and histological character of the tumor.

	serum VEGF-A concentration [pg/ml] before surgery
**Grade of malignancy according to WHO**	0.0690
**Tumor malignancy (malignant/non-malignant)**	-0.0508

VEGF-A, vascular endothelial growth factor—A.

The values of serum VEGF-A do not allow confirmation of the assumption—in the case of the WHO scale, strength of the relationship was low, and although consistent, not statistically significant. In the dichotomous division (malignant / non-malignant) the relationship was also negligible, but in this case it was opposite and not statistically significant. The lack of statistical significance indicates that this direction is random.

In order to refine the analysis, the differentiation was performed on the basis of tumor type (according to the classification of 5 tumor types: meningiomas, LGG, HGG, metastatic tumors, other). An analysis of the differences in pre-surgery serum VEGF-A concentration due to the type of tumor, was performed. Data on differentiation are presented in Table 6.

**Table 6:** Kruskal-Wallis ANOVA rank for serum VEGF-A concentration [pg/ml] before surgery and tumor type, Kruskal-Wallis test: H (3, N = 48) = 1.047748 p = .7897.

Type of tumor (by 5 general groups)	N—valid	Rank
**Metastatic tumors**	6	119.0
**Meningiomas**	9	221.5
**Other**	3	64.0
**LGG**	7	196.5
**HGG**	23	575.0

LGG, low grade gliomas; HGG, high grade gliomas.

**Fig 1:**
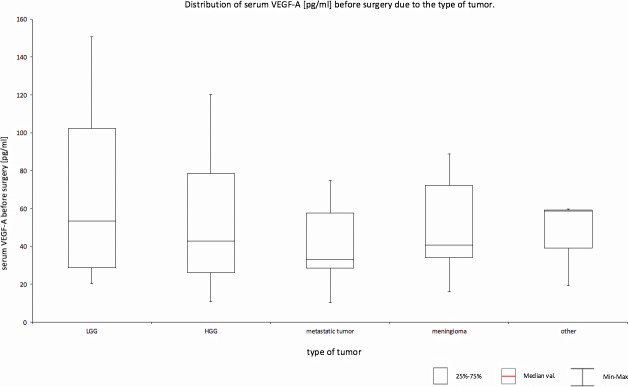
Distribution of serum VEGF-A [pg/ml] before surgery due to the type of tumor.

Despite the lack of significant differences, their distributions were determined (Fig 1). People with cancer identified as "other" had the highest median results (58.77), with the lowest index spread (20–60) at the same time. The highest variability was found in the glioblastoma group—twice as large as in meningiomas or metastatic tumors, and affects both HGG and LGG types. Metastatic tumors were characterized by the lowest median position and the smallest stretch of results.

Based on the analysis of the results, in the second step, the association between serum VEGF-A concentration and tumor histologic type was not confirmed.

## Discussion

This study has confirmed that before surgery the serum VEGF-A concentration in patients with central nervous system tumors is higher than in non-cancer patients. In the study group the concentration range ranged from 10.39 to 150.57 pg/ml (median 41.70 pg/ml), while in the comparative group 0.00–130.77 pg/ml (median 22.56 pg/ml). Therefore, a higher serum VEGF-A concentrations, in patients with intracranial tumors, may be expected to be associated with active neoangiogenesis within the tumor. In addition it can be confirmed by the high strength and consistent correlation between serum VEGF-A before and after surgery, where the one before surgery was noted as higher.

Rafat et al. [5] studied 22 patients with brain tumors (12—GM, glioblastoma multiforme; 10 metastatic tumors) and 10 healthy volunteers. Serum VEGF concentrations in patients with GM (before brain tumor surgery) and metastatic tumors were significantly higher than in control group (p <0.0001).

Chiorean et al. [6] also examined preoperative plasma VEGF levels in patients with diagnosed glioblastoma multiforme. The study enrolled a group of 14 patients and 32 people from the control group, without tumor diagnosis. The serum VEGF concentration was determined by ELISA. Median for the study group was 300 ng/ml (284–374 ng/ml) and 104 ng/ml for the control group (80–141 ng/ml). There was no statistically significant correlation between serum VEGF levels and overall survival.

Yang et al. [7] studied the expression of VEGF by ELISA in patients with glioma. The pre-surgery VEGF level in the study group was significantly higher than in the control group (without tumor diagnosis). The VEGF concentration in the study group decreased significantly after the surgery, compared to the one before surgery (P <0.05). The expression level of VEGF correlated with histological glioma type, it’s malignancy according to WHO, metastasis and overall survival (P <0.05). These results suggested that VEGF may play a key role in the development of glioma.

The difference in serum concentration of this factor was also demonstrated by Crocker [8]. In his work he studied serum levels of angiogenic factors, including VEGF-A, among 36 patients with glioblastoma and 5 of the control group. Range of concentrations in the study group was 136–793 pg/ml and in the control group 23–409 pg/ml. The pre-surgery concentration of VEGF-A was higher than the one after surgery. The author concluded that higher VEGF-A concentration in patients with intracranial tumors is associated with ongoing tumor’s active vasculogenesis.

Studies of the presence of VEGF in cerebrospinal fluid in patients with glioblastoma, Peles et al. [9] demonstrated that mean VEGF level was significantly higher in patients with high grade glioma compared to patients with low grade glioma. VEGF concentrations were 17.6, 7.2 and 8.3 ng/ml respectively, P <0.005. Elevated levels of angiogenic factors in cerebrospinal fluid correlated with tumor vasculature and were unfavorable for patient overall survival. Serum VEGF concentration did not correlate with malignancy, tumor vascularization nor overall survival.

In this study we have not been able to confirm the association between serum VEGF-A concentration and malignancy and histological type of intracranial tumor. In the case of a four-grade tumor malignancy scale, the tested strength of the relationship was small, and although consistent, it was statistically insignificant. In the case of the histological type, there were also no statistically significant differences. The highest pre-surgery serum VEGF-A concentration was found in patients with low grade gliomas, slightly lower (similar to each other) levels with high grade glioblastomas and meningiomas, while the lowest level was characteristic for metastatic tumors. Characteristic was also high variability in LGG patients (52.56 pg/ml), and was higher than that the one reported in patients with HGG (32.38 pg/ml). In the rest types of tumors the differentiation was similar and oscillated within 23.08–27.50 pg/ml.

## Conclusions

Based on the obtained results and statistical analyzes, the following conclusions were drawn:

1. In patients with central nervous system tumors, serum VEGF-A concentration is higher than in patients without tumor diagnosis.
2. In patients with central nervous system tumors, pre-surgery serum VEGF-A concentration is higher than post-surgery concentration.
3. Determination of serum VEGF-A concentration in patients with central nervous system tumors, and comparing them with routine examinations can translate into more accurate choice of treatment and evaluation of patient’s overall survival.
4. Evaluation of the neoangiogenesis process can be clinically relevant in determining prognosis, treatment planning, and monitoring of central nervous system tumors.
